# Result of a year-long animal survey in a state-owned forest farm in Beijing, China

**DOI:** 10.3897/BDJ.10.e91132

**Published:** 2022-10-05

**Authors:** Xiangying Shi, Ge Sun, Xinyu Yang, Junhong Gao, Lingdi Tan, Yuexin Song, Yiping Hu, Zunxiu Zhou, Huibin Zhao, Zhihai Hu, Shunwan Zhu, Yufan Cao, Rui Liao, Wei Chen, Zhehao Wu, Xiangyu Guan, Xiaotong Ren, Shen Zhang

**Affiliations:** 1 College of Environmental Sciences and Engineering, Peking University, Beijing, China College of Environmental Sciences and Engineering, Peking University Beijing China; 2 Shan Shui Conservation Center, Beijing, China Shan Shui Conservation Center Beijing China; 3 Ecology and Nature Conservation Institute,Chinese Academy of Forestry, Beijing, China Ecology and Nature Conservation Institute,Chinese Academy of Forestry Beijing China; 4 Beijing Forestry Carbon Administration, Beijing, China Beijing Forestry Carbon Administration Beijing China; 5 Jingxi Forest Farm, Beijing, China Jingxi Forest Farm Beijing China; 6 Sichuan Academy of Forestry, Chengdu, China Sichuan Academy of Forestry Chengdu China; 7 Center for Nature and Society, College of Life Sciences, Peking University, Beijing, China Center for Nature and Society, College of Life Sciences, Peking University Beijing China; 8 Mountain Cats Culture Communication Co., Ltd, Beijing, China Mountain Cats Culture Communication Co., Ltd Beijing China; 9 Guanxiangyu Ecological Technology Co. Ltd, Beijing, China Guanxiangyu Ecological Technology Co. Ltd Beijing China; 10 Institute of Ecology, College of Urban and Environmental Sciences, Peking University, Beijing, China Institute of Ecology, College of Urban and Environmental Sciences, Peking University Beijing China

**Keywords:** biodiversity, mammals, birds, reptiles, amphibians, fishes, insects, artificial forest, natural forest, Jingxi Forest Farm

## Abstract

Background

Artificial forest can have great potential in serving as habitat to wildlife, depending on different management methods. As the state-owned forest farms now play a new role in ecological conservation in China, the biological richness of this kind of land-use type is understudied. Once owned by a mining company, a largest state-owned forest farm, Jingxi Forest Farm, has been reformed to be a state-owned forest farm with the purpose of conservation since 2017. Although this 116.4 km^2^ forest farm holds a near-healthy montaine ecosystem very representative in North China, a large proportion of artificial coniferous forest in the forest farm has been proven to hold less biodiversity than natural vegetation. This situation, however, provides a great opportunity for ecological restoration and biodiversity conservation. Therefore, from November 2019 to December 2020, we conducted a set of biodiversity surveys, whose results will serve as a baseline for further restoration and conservation.

New information

Here, we report the result of a multi-taxa fauna diversity survey conducted in Jingxi Forest Farm mainly in year 2020 with explicit spatial information. It is the first survey of its kind conducted in this area, revealing a total of 19 species of mammals, 86 birds, four reptiles, two amphibians and one fish species, as well as 101 species of insects. Four species of mammals are identified as data-poor species as they have less than 100 occurrence records with coordination in the GBIF database. One species of insect, representing one new provincial record genus of Beijing, is reported.

## Introduction

A large effort on reforestation and afforestation has been made in China, constituting nearly one fourth of the global growth in forest as from year 2000 to 2017 as an example, which has been suggested as a great opportunity for biodiversity conservation (Hua et al. 2016, Chen et al. 2019). However, there are very few considerations of biodiversity effects of these projects, with monoculture species, high density stems and low underlayer coverages. Monoculture forests have been proven to support less biodiversity, as well as other critical ecosystem services, such as water yield and soil erosion control, than natural forests, both old-growth and natural-recovered (Wang et al. 2019, Ke et al. 2020, Hua et al. 2022). Therefore, it is necessary to set up new plans for transforming the existing artificial forest to more natural and complex ones, as well as implementing reforestation with a better design, including natural and assisted natural regeneration, as a crucial part for further ecological restoration.

State-owned forest farms are government entities that manage state-owned forest and related land in China (Ni et al. 2022). There are 64.7 million hectares of forestlands in China managed by state-owned forest farms up to 2019, consisting of 22.8% of forestlands of the country (National Forestry and Grassland Administration 2021). With the "Natural Forest Protection Policy" and other forest policies, timber logging is strictly limited national wide; therefore, most forest farms now serve with a new role in nature conservation and recreation. Therefore, there is a great potential to estimate and improve biodiversity conservation in the artificial forests of forest farms. However, earlier studies on forest farms mainly focused on single species groups or other environmental indicators (Kwok and Corlett 2000, Liang et al. 2013, Wang et al. 2019).

In this study, we provide the first comprehensive inventory of the largest state-owned forest farm in Beijing, the Jingxi Forest Farm, which is a representative of the forest ecoysystem of North China Taihang Mountain Range. The dataset results from our multi-species approach including mammals, birds and insects. The data would be a baseline to be provided for a biodiversity restoration pilot project by the Beijing City government, that restoration intervention and management would improve the biodiversity function of existing artifical forests.

## Material and methods

### Study site

The study site, Jingxi Forest Farm (39.94N, 115.81E) is located in the west of the Municipality of Beijing, the capital city and one of the mega-cities in the world with ca. 25 million residents. This state-owned forest farm is by far the largest one of its kind in Beijing, with an area of 116.4 km^2^. It used to belong to the Beijing Jingmei Group, a state-owned coal mine company and a large proportion of its artificial forest was planted for industrial use since 1970s. The whole forest farm was transferred under the management of Beijing Municipal Forestry and Parks Bureau in 2017; therefore, all forests were then managed for the ecological purpose of public interest alone. The key responsiblilty of the forest farm management includes tending and thinning of existing forests, ecological restoration on former mining area by reforestation, fire prevention and patrolling against poaching. No other human activity is officially allowed so far, although some level of fungi collecting and hiking may occur. This area also serves an important role in preserving connectivity from the great mountains of of the centre Taihang where leopard (*Pantherapardusjaponesis*) still exists (Luo et al. 2020) to the North-China Plain where tens of millions of people live.

Most part of the Jingxi is mountain area, ranging from 200 m a.s.l. to 1610 m a.s.l. with a temperate continental climate. According to the management office, this site has a mean annual temperature of 7-10°C and mean annual precipitation around 600 mm. It has a mixed vegetation of artificial and natural secondary forest on the shady slopes, as well as scrublands on most of the sunny slopes, except for the artificial forest on less steep sunny surfaces. As of 2017, Jingxi has 36.3 km^2^ of forest with canopy coverage above 20%, 63% of the area of which is pure coniferous forest, all artificially originated. The remaining part is a mixture of artificial and secondary natural forests, consisting of broadleaved or mixed forests. With rising elevation, the main tree species in artificial coniferous forest change from *Pinustabuliformis* to Larixgmeliniivar.principis-rupprechtii, with occasionally naturally-growing *Ulmusparvifolia* and Fraxinuschinensissubsp.rhynchophylla individuals. Meanwhile, the foundation species in secondary natural forests changes from Populustremulavar.davidiana to Betulapendulasubsp.mandshurica, with *Quercusmongolica* being seen within natural forests at all elevations. This area shared some of its western boundaries with Beijing Baihua Mountain National Reserve, resulting a similar composition in flora community (Zhang et al. 2020).

### Sampling design and methods

Our work mainly aimed at collecting fauna biodiversity information for this newly-established forest farm, providing a baseline for further management and planning. We undertook a systematic survey on several animal taxa, including mammals (camera traps and mouse traps, thus no bats were sampled), birds (visual observation and vocal identification on transects at dawn, usually within 3 hours after sunrise; for detailed survey time and effort, see Suppl. material 1; no nocturnal species were noticed) and insects (pitfall traps for quantitative surveying Carabidae species, Malaise trap for more general species), while using convenience sampling on other vertebrates and other taxa of insects during daytime (mainly Odonata and Papilionoidea). The project was started in late 2019 and is still in progress; and here we are reporting results mostly up to 31 Dec 2020, before all restoration measurement took place. For different taxa and methods, spatial and temporal coverage may vary.

**Figure 1.**
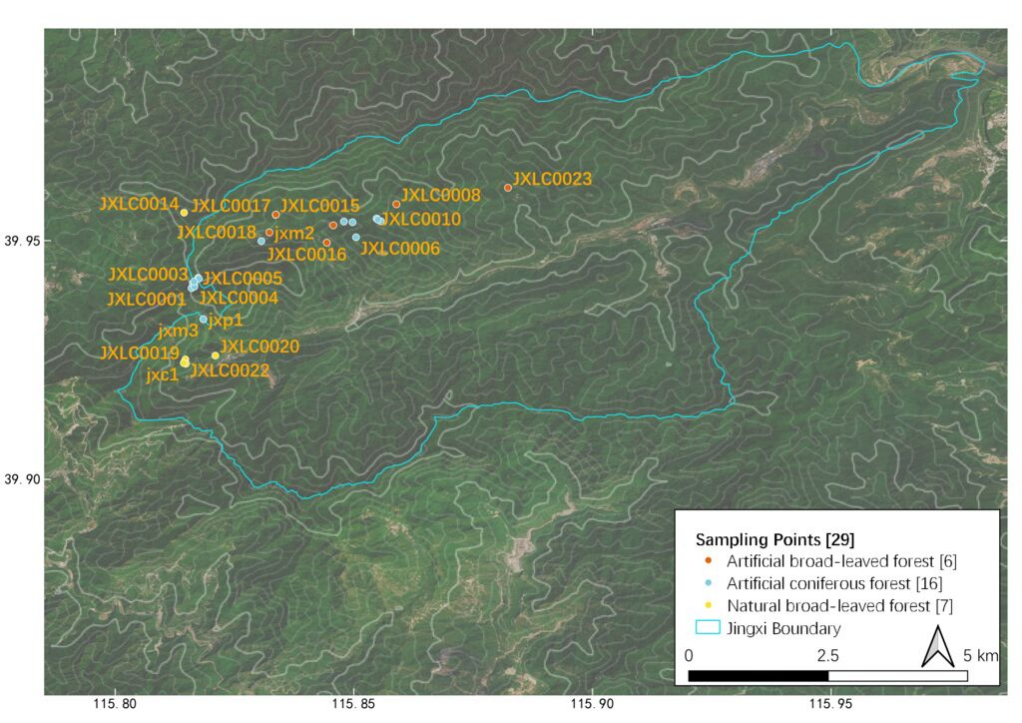
Sampling points and their distribution in Jingxi, see Table 2. Contour lines representing elevation, using data from Earth Resources Observation and Science (EROS) Center, USGS (2018).

**Figure 2.**
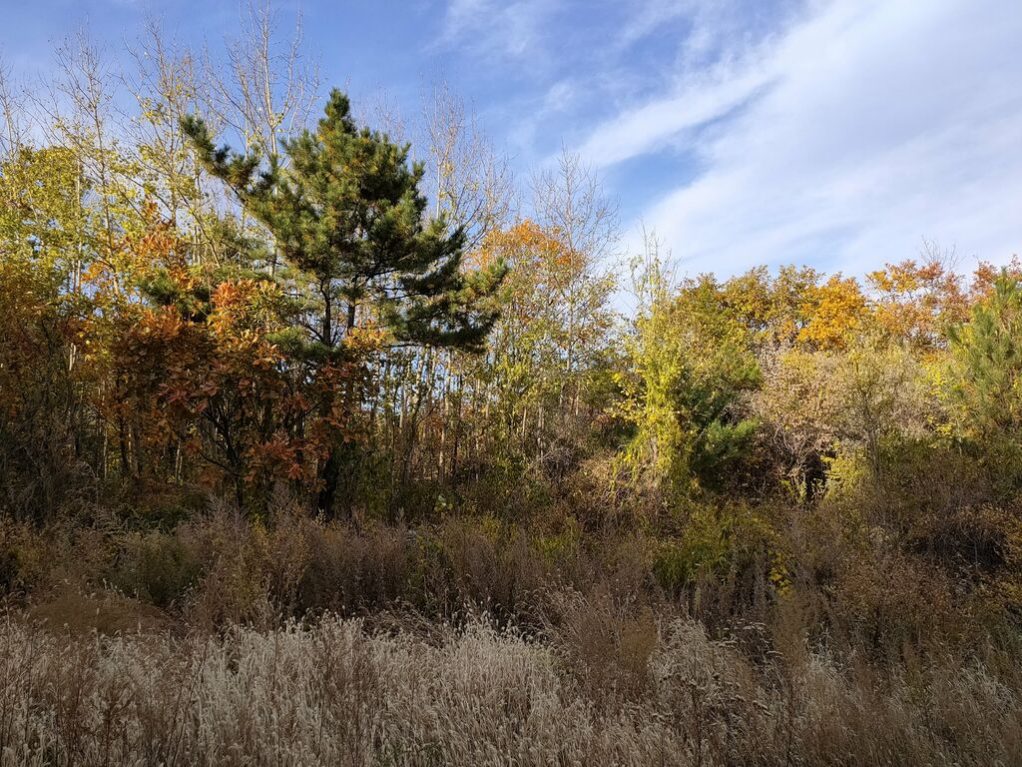
Natural secondary deciduous broad-leaved forest in Jingxi (photo taken in October), mainly a mixed community consisting of *Quercusmongolica* and Populustremulavar.davidiana.

**Figure 3.**
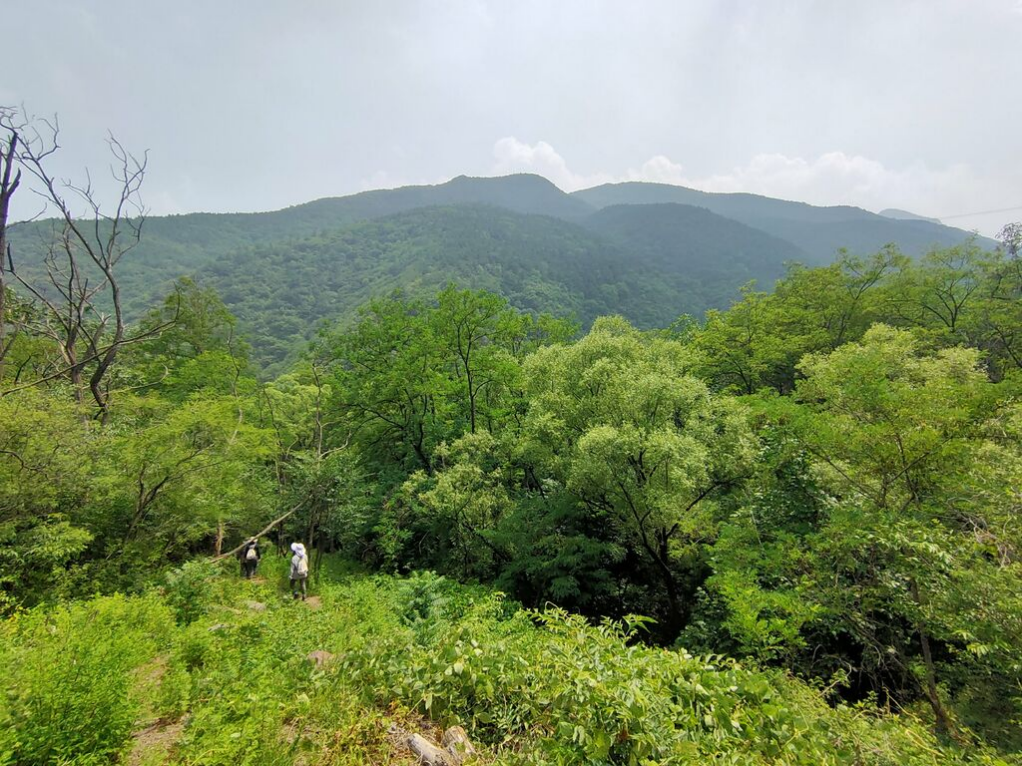
Artificial deciduous broad-leaved forest (photo taken in June), including *Robiniapseudoacacia*, *Juglansregia*, *Crataeguspinnatifida* and other commonly cultivated tree species.

**Figure 4.**
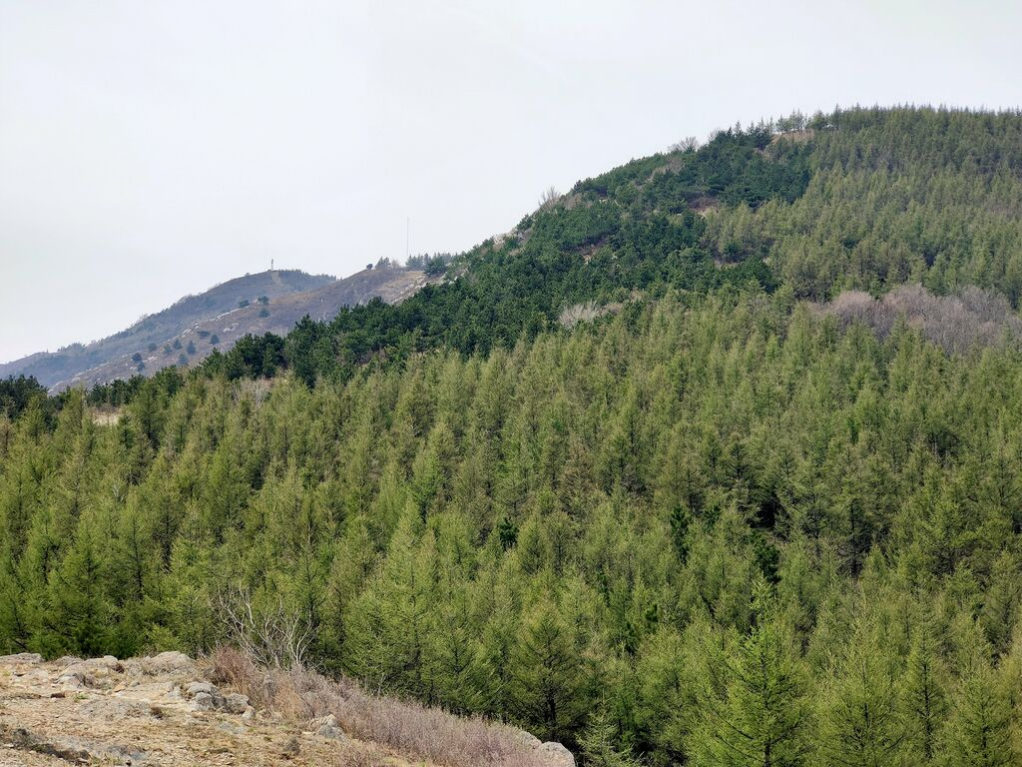
Artificial coniferous forest (photo taken in April), first planted in 1970s, generally *Pinustabuliformis* or Larixgmeliniivar.principis-rupprechtii pure forest or a mixture of both species.

**Figure 5.**
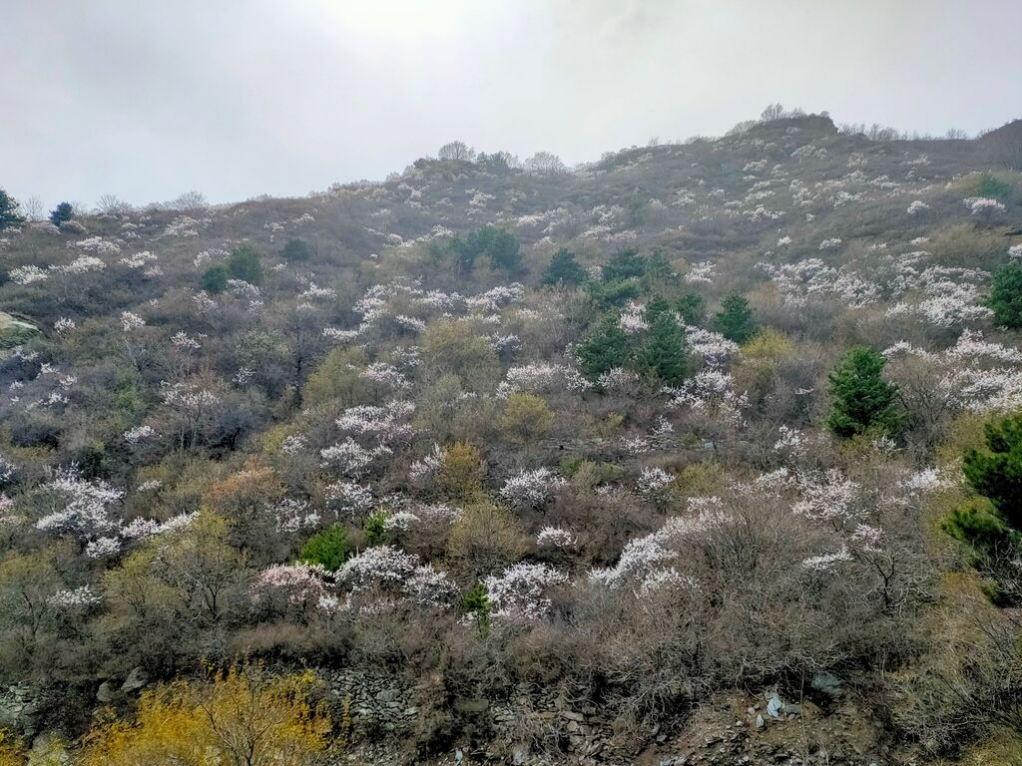
Shrub community on sunny slopes (photo taken in April), constituting of Vitexnegundovar.negundo, Ziziphusjujubavar.spinosa, *Prunusdavidiana*, *Prunussibirica* and other species.

Details of sampling methods, efforts and date coverage are listed in Table 1, while spatial distribution of sampling points is listed in Table 2 (Fig. 1). As the whole area had various vegetation types, we recorded the vegetation type at each sampling point. Abbreviation of forest types in Table 2: NB = natural secondary deciduous broad-leaved forest, mainly a mixture community consisting of *Quercusmongolica* and Populustremulavar.davidiana at our sampling points (*Fig. 2*); AB = artificial deciduous broad-leaved forest, usually near abandoned (from 1970s) villages, including *Robiniapseudoacacia*, *Juglansregia*, *Crataeguspinnatifida* and other commonly cultivated tree species in Beijing (Fig. 3); AC = artificial coniferous forest, first planted in 1970s, mainly *Pinustabuliformis* or Larixgmeliniivar.principis-rupprechtii pure forest or a mixture of both species (Fig. 4). We did not sample in any scrub habitats (Fig. 5), which are abundant on most steep sunny slopes, but hard to approach for both human and other medium-large mammals, except *Naemorhedusgriseus*.

## Data resources

Our survey records 19 species of mammals, 86 birds, four reptiles, two amphibians and one fish, as well as 101 species of insects. By far, we are only reporting occurrence information here and this information does not represent any absence data.

All mammals recorded, but one species (Table 3, Table 4) are listed as Least Concern in the IUCN Red List (IUCN 2021), with *Naemorhedusgriseus* previously listed as Vulnerable (Duckworth et al. 2008, see discussion). Two Sepcies (*Prionailurusbengalensis* and *Naemorhedusgriseus*) are listed as Vulnerable in China's Red List of Biodiversity (Jiang 2021), as well as China’s Key Protected Wild Animals as 2^nd^ level protected animal. Four species have less than 100 occurrence records with coordination in GBIF database, therefore identified as data-poor species.

Birds are only reported as checklists (Table 5) from both transects, more detailed records have been uploaded to eBird database and can be downloaded for further analysis (Suppl. material 1). None of the species is Threatened species according to the IUCN Red List, seven species are listed as NT in China's Red List of Biodiversity (Zhang and Zheng 2021), while 14 species are listed in China’s Key Protected Wild Animals as 2^nd^ level protected animal.

Other vertebrates are mostly recorded, based on convenience sampling and are listed in Table 6. One sepcies (*Elaphecarinata*) is listed as Endangered in China's Red List of Biodiversity (Wang et al. 2021, Jiang et al. 2021) with other species listed as Least Concern. There are two main streams in the area and we sampled one for fish and amphibians. Reptiles were recorded on encounter.

A checklist of insects are also reported with our method of survey (Table 7). One species representing one new provincial record genus of Beijing (*Claddiscusobeliscus* Lewis, 1895) is reported.

## Results

The total number of 213 species of animals were recorded in Jingxi area by the end of the year 2020. Amongst them, one species was listed as EN and two species were listed as VU in China's Red List of Biodiversity. More species of mammals (natural forest: 14, in total 19; artifical coniferous forest: 11, in total 19), birds (natural forest: 75, in total 86; artifical coniferous forest: 69, in total 86) were recorded in natural forest than in artificial coniferous forests. No insect sampling was done in natural forest, so no comparison was available.

## Discussion

Limited by our human power and project design, we did not conduct systemtic sampling all over the site. Additionally, some of the sampling points lie in close vicinity with each other, so occurrence data from these points may not be independent. It is advised that great caution should be taken when accounting our occurrence data into any further modelling. However, comparing to exsiting GBIF data, our occurrence data, as well as environment information still expand the knowledge of several data-poor species in this less-studied area and add evidence to the comparison of biodiversity between artificial forest and natural forests (Carnus et al. 2006, Brockerhoff et al. 2008, Horák et al. 2019, Wang et al. 2019).

Our data show that there is a clear trend that artificial coniferous forests hold less mammalian and avian biodiversity than natural forest of the same age, altitude and slope, even with more sampling effort, larger coverage (see Table 2) in artificial forests. This justifies our suggestion that artificial coniferous forests should be modified for better biodiversity and other ecosystem services outcomes. Our result also suggest that, despite large areas of artifical forest present, these sites still server as important wildlife habitats, as other artificial forests can do (Harich and Treydte 2016, Tanalgo et al. 2021). Jingxi supports biodiversity similar to one important nature reserve, Baihua Mountain National Nature Reserve, which lies in the close vicinity to the west of Jingxi. The NNR have better natural vegetation (Zhang et al. 2020) and further distance to the urban area, thus less human disturbance, comparing to Jingxi; thus, there is a good opportunity for comparing biodiversity on different spots in the names of different vegetation, human disturbance and management planning. As an example, comparing with latest camera trap report and historical checklist from Baihua Mountain NNR (Fu et al. 1994, Liu et al. 2018), we reported a nearly similar composition of mammal species, except for small mammals like bats, rodents and shrews. This similarity in mammal composition shows that Jingxi has a great importance in providing suitable and specific habitats and connections for wildlife.

Our results also call for surveying and monitoring projects to take place in nearby areas and their results to be published with clear spatial information. Although not rarely captured by camera traps in our study, four over 19 total recorded mammal species are data-poor in GBIF, indicating great potential of occurrence data to be published. Furthermore, monitoring of the population trend of vulnerable species *N.griseus* at larger scale is crucial, as it was once listed as VU in the IUCN Red List, while it is not currently assessed in the Red List as Mori et al. (2019) suggested that *N.griseus* should be treated as a subspecies of *N.goral*. The lack of assessment and information could become a barrier in the conservation of this nationally important and vulnerable species/population. Therefore, more information and long-term monitoring on this population is important for further conservation actions.

Finally, there are still knowledge gaps on information about amphibians and reptiles, as well as bats in vertebrates, while no flying squirrels were witnessed within the area. No research on either amphibian or reptile diversity was found for this area or adjoining areas like Baihua Mountain NNR. With a total of five species of amphibians and reptiles beingreported in Jingxi, it shows great potential for a systematic survey to be conducted, comparing with the result of 22 species municipality-wide (Shi et al. 2022). Additionally, we still have a poor level of different taxa of insects covered in our survey as only pitfall traps and malaise traps were used, while it is still more challenging when it comes to other invertebrate taxa.

## Conclusions

This dataset shows that an artificial forest farm near a mega city such as Beijing can harbour a considerable biological richness and serve as natural habitat to wildlife. It adds evidence to findings from other forest types with a multi-species approach (Kwok and Corlett 2000, Stephens and Wagner 2007, Wang et al. 2019). This is also the first report (both in English and Chinese) on biodiversity in Jingxi. Our results provided a baseline for a next step of ecological restoration, while showing a trend that natural forest held all and more species than in artificial forests on site, justifying our design that transforming artificial forest to natural forest as a means of the restoration.

Considering the large proportion of artificial habitat, especially pure coniferous forest, presenting in Jingxi, it is also worth implementing further management on plant community, based on our knowledge of fauna species’ preference, promoting richer biodiversity and more lively environment near the urban community.

## Supplementary Material

37D98F6F-445E-5F75-93C5-0669F0256C92 10.3897/BDJ.10.e91132.suppl1 Supplementary material 1 S1 List of eBird checklists at Jingxi Data type checklists Brief description a List of eBird checklists at Jingxi File: oo_671719.txt https://binary.pensoft.net/file/671719 Zhang, Shen

## Figures and Tables

**Table 1.** Sampling methods, efforts and date coverage.

Taxon	Method	Sampling effort	Sampling time	Identify references	Nomenclature	Id experts
Mammals	camera trap, EREAGLE® E1C & E3H	22 sites/cameras	29 Nov. 2019 - 31 Dec. 2020	Chen et al. (2002)	Jiang et al. (2017)	Sun G., Hu Y.
Small Mammals (Rodents and Shrews)	mousetrap with sunflower seed	3 sites, 200 traps * 1 night	19 Aug. 2020 - 20 Aug. 2020		Jiang et al. (2017)	Liao R.
Birds	transect survey at dawn, usually finishes within 3 hours after sunrise; visual (with 8*42 binoculars) and vocal identification	2 transects, 500 m each, detailed sampling date see Suppl. material 1	18 Apr. 2020 - 24 Oct. 2020	MacKinnon et al. (2000), Zhao (2018), recordings on xeno-canto.org	Zheng (2017)	Huang H., Wu Z., Zhang S.
Other Vertebrate	Convenience sampling by observation	NA	29 Nov. 2019 - 31 Dec. 2020		Taxonomy of reptiles and amphibians follows Wang et al. (2020). Fishes see Zhang and Zhao (2013)	Wu Z., Qi S.
Insects	Townes type Malaise trap, 1.8 m length * 1.2 m width, 2 m middle ridge	1 trap	20 May 2020 - 26 Aug. 2020	Chen et al. (1959), Raske (1973), Tan and Yu (1980), Pu (1980), Zhao and Chen (1980), Jiang et al. (1985), Tian and Yu (1994), Kryzhanovskij et al. (1995), Yu (1995), Yu (1996), Ren et al. (1998), Zhang et al. (1998), Tian (2000), Ren and Li (2001), Jiang (2002), Růžička (2002), Yu et al. (2002), Kim and Kim (2003), Huang and Jiang (2004), Yu et al. (2004), Han et al. (2005), Fan et al. (2007), Jung and Kim (2009), Ren et al. (2009), Yu (2010), Yang and Ren (2011), Ji (2012), Zahradník (2012), Chen (2013), Hu et al. (2013), Warren-Thomas et al. (2014), Zhao (2014), Zhou et al. (2014), Lin (2015), Liu et al. (2015), Yu et al. (2016), Wei (2017), Xu et al. (2018), Bai et al. (2019), Egorov (2019), Hui (2019), Yu (2020), Fabrizi et al. (2021)		Huang Z.
Insects	pitfall trap, ø = 8 cm, d = 13.5 cm, filled with saturated NaCl solution	24 traps	20 May 2020 - 1 Oct 2020	Same as above.		Huang Z.
Insects	Convenience sampling by observation	NA	29 Nov. 2019 - 31 Dec. 2020	Zhang (2018), Wu and Xu (2017)		Chen W.

**Table 2.** Sampling points and its environment.

Method	ID	latitude/°	longitude/°	elevation/m	vegetation
camera trap	JXLC0001	39.94007	115.81598	1012	AC
camera trap	JXLC0002	39.94079	115.81655	1022	AC
camera trap	JXLC0003	39.94105	115.81627	1040	AC
camera trap	JXLC0004	39.94037	115.81657	1027	AC
camera trap	JXLC0005	39.94222	115.81736	989	AC
camera trap	JXLC0006	39.95071	115.85044	1014	AC
camera trap	JXLC0008	39.95763	115.85891	725	AB
camera trap	JXLC0009	39.95404	115.85568	767	AC
camera trap	JXLC0010	39.95463	115.85475	784	AC
camera trap	JXLC0011	39.95400	115.84791	772	AC
camera trap	JXLC0012	39.95386	115.84971	800	AC
camera trap	JXLC0013	39.95321	115.84568	712	AB
camera trap	JXLC0014	39.95586	115.81442	707	NB
camera trap	JXLC0015	39.95541	115.83362	626	NB
camera trap	JXLC0016	39.94957	115.84432	1014	AB
camera trap	JXLC0017	39.95545	115.83365	775	AB
camera trap	JXLC0018	39.94990	115.83061	850	AC
camera trap	JXLC0019	39.92456	115.81445	925	NB
camera trap	JXLC0020	39.92594	115.82098	924	NB
camera trap	JXLC0021	39.94203	115.81749	942	AC
camera trap	JXLC0022	39.92515	115.81470	897	NB
camera trap	JXLC0023	39.96107	115.88228	553	AB
Malaise trap	jxmt	39.95440	115.85500	784	AC
mouse trap	jxm1	39.92422	115.81481	900	NB
mouse trap	jxm2	39.95172	115.83231	729	AB
mouse trap	jxm3	39.93356	115.81845	858	AC
transect	jxt1	39.94142	115.81650	950	AC
transect	jxc1	39.92437	115.81435	950	NB
pitfall trap point	jxp1	39.93356	115.81845	858	AC

**Table 3.** Mammals and their point-level occurrence, with "y" illustrating detection in this site.

Species	*Crocidurashantungensis*	*Erinaceusamurensis*	*Mustelasibirica*	*Melesleucurus*	*Arctonyxcollaris*	*Pagumalarvata*	*Prionailurusbengalensis*	*Lepustolai*	*Sciurusvulgaris*	*Sciurotamiasdavidianus*
China’s Key Protected Wild Animals							II			
China's Red List of Biodiversity	LC	LC	LC	NT	NT	NT	VU	LC	NT	LC
Number of occurrence records with coordination in GBIF (if n < 100)					52 (GBIF Secretariat 2021a)					64 (GBIF Secretariat 2021b)
JXLC0001				y				y	y	
JXLC0002				y				y	y	
JXLC0003				y	y		y	y	y	
JXLC0004				y			y	y		
JXLC0005				y						
JXLC0006								y	y	
JXLC0008				y		y	y			y
JXLC0009										
JXLC0010				y			y	y		y
JXLC0011								y		y
JXLC0012				y				y		y
JXLC0013				y	y	y	y	y	y	
JXLC0014				y	y	y	y			y
JXLC0015				y	y		y	y		y
JXLC0016							y			y
JXLC0017		y	y	y	y		y	y	y	y
JXLC0018							y			y
JXLC0019				y				y	y	
JXLC0020				y			y	y	y	y
JXLC0021								y	y	
JXLC0022				y			y	y	y	
JXLC0023				y		y	y			y
jxm1										
jxm2										
jxm3	y									
visiual observation at daytime								y	y	y

**Table 4.** Continued: Mammals and their point-level occurrence, with "y" illustrating detection in this site.

species	*Tamiassibiricus*	*Tamiopsswinhoei*	*Apodemuspeninsulae*	*Apodemusagrarius*	*Niviventerconfucianus*	*Tscherskiatriton*	*Susscrofa*	*Capreoluspygargus*	*Naemorhedusgriseus*
China’s Key Protected Wild Animals									II
China's Red List of Biodiversity	LC	LC	LC	LC	LC	LC	LC	NT	VU
Number of occurrence records with coordination in GBIF (if n < 100)						76 (GBIF Secretariat 2021c)			15 (GBIF Secretariat 2021d)
JXLC0001					y			y	
JXLC0002							y	y	
JXLC0003									
JXLC0004								y	
JXLC0005								y	
JXLC0006									
JXLC0008		y						y	
JXLC0009							y	y	
JXLC0010					y		y		
JXLC0011							y	y	
JXLC0012	y				y		y		
JXLC0013							y	y	y
JXLC0014							y		y
JXLC0015							y	y	y
JXLC0016									y
JXLC0017							y	y	
JXLC0018					y				
JXLC0019							y	y	
JXLC0020					y		y	y	
JXLC0021								y	
JXLC0022							y	y	
JXLC0023	y				y			y	
jxm1			y	y	y	y			
jxm2			y	y	y				
jxm3					y				
visiual observation at daytime	y	y					y	y	y

**Table 5.** Checklists of birds with their occurrence in two transects in Jingxi, with their protection level in China.

Bird species	China's Key Protected Wild Animals	China's Red List of Biodiversity	Number of occurrences in jxt1	Number of occurrences in jxc1
*Pucrasiamacrolopha*	II	LC	0	4
*Phasianuscolchicus*		LC	9	8
*Anascrecca*		LC	0	1
*Streptopeliaorientalis*		LC	1	1
*Hirundapuscaudacutus*		LC	0	1
*Apusapus*		LC	0	1
*Hierococcyxsparverioides*		LC	2	4
*Cuculusmicropterus*		LC	0	1
*Cuculussaturatus*		LC	1	2
*Turnixtanki*		LC	0	1
*Pernisptilorhynchus*	II	NT	2	1
*Accipitergularis*	II	LC	1	1
*Accipiternisus*	II	LC	1	3
*Circusspilonotus*	II	NT	0	1
*Circuscyaneus*	II	NT	0	1
*Circusmelanoleucos*	II	NT	0	1
*Milvusmigrans*	II	LC	1	0
*Dendrocoposcanicapillus*		LC	2	1
*Dendrocoposmajor*		LC	1	1
*Picuscanus*		LC	0	1
*Falcotinnunculus*	II	LC	1	1
*Falcosubbuteo*	II	LC	1	1
*Falcoperegrinus*	II	NT	0	1
*Pericrocotusethologus*		LC	0	1
*Dicrurushottentottus*		LC	1	0
*Garrulusglandarius*		LC	3	4
*Urocissaerythroryncha*		LC	2	3
*Picapica*		LC	3	4
*Pyrrhocoraxpyrrhocorax*		LC	2	0
*Corvusmacrorhynchos*		LC	5	7
*Periparusater*		LC	2	1
*Pardaliparusvenustulus*		LC	7	8
*Poecilepalustris*		LC	3	7
*Poecilemontanus*		LC	10	11
*Paruscinereus*		LC	9	10
*Alaudaarvensis*	II	LC	1	0
*Locustellatacsanowskia*		LC	0	1
*Pycnonotussinensis*		LC	2	3
*Phylloscopusfuscatus*		LC	0	2
*Phylloscopusarmandii*		LC	6	7
*Phylloscopusschwarzi*		LC	2	3
*Phylloscopusyunnanensis*		LC	4	2
*Phylloscopusproregulus*		LC	5	4
*Phylloscopusinornatus*		LC	3	3
*Phylloscopushumei*		LC	1	0
*Phylloscopusborealis*		LC	1	2
*Phylloscopusplumbeitarsus*		LC	4	3
*Phylloscopuscoronatus*		LC	2	3
*Phylloscopusclaudiae*		LC	7	7
*Hororniscanturians*		LC	6	6
*Urosphesquameiceps*		LC	3	7
*Aegithalosglaucogularis*		LC	10	7
*Rhopophiluspekinensis*		LC	5	11
*Sinosuthorawebbia*		LC	4	7
*Zosteropserythropleurus*	II	LC	0	1
*Zosteropsjaponicus*		LC	2	4
*Garrulaxdavidi*		LC	8	9
*Sittavillosa*		NT	6	3
*Turdusruficollis*		LC	1	0
*Turdusnaumanni*		LC	1	0
*Turdusmupinensis*		LC	3	4
*Larvivoracyane*		LC	4	2
*Calliopecalliope*	II	LC	1	1
*Tarsigercyanurus*		LC	2	1
*Phoenicurusauroreus*		LC	6	9
*Muscicapasibirica*		LC	1	0
*Ficedulazanthopygia*		LC	2	2
*Ficedulaelisae*		NT	2	2
*Ficedulaalbicilla*		LC	0	2
*Regulusregulus*		LC	1	0
*Prunellacollaris*		LC	1	0
*Prunellamontanella*		LC	1	1
*Passercinnamomeus*		LC	2	0
*Motacillatschutschensis*		LC	1	2
*Motacillaalba*		LC	1	3
*Anthusrichardi*		LC	0	2
*Anthushodgsoni*		LC	3	3
*Fringillamontifringilla*		LC	2	1
*Carpodacuserythrinus*		LC	4	2
*Carpodacusdavidianus*		LC	5	6
*Chlorissinica*		LC	3	5
*Spinusspinus*		LC	1	1
*Emberizagodlewskii*		LC	8	10
*Emberizacioides*		LC	7	2
*Emberizapusilla*		LC	3	2
*Emberizaelegans*		LC	1	1
Number of species in total	14	86	69	75

**Table 6.** Checklist of other vertebrates and coordinates of their occurrence records.

Class	Order	Family	Species	China's Red List of Biodiversity	Latitude	Longitude
Actinopterygii	Cypriniformes	Leuciscidae	*Rhynchocyprislagowskii*	LC	39.9517	115.8410
Amphibia	Anura	Bufonidae	*Bufogargarizans*	LC	39.9517	115.8410
Amphibia	Anura	Ranidae	*Ranachensinensis*	LC	39.9517	115.8410
Reptilia	Squamata	Colubridae	*Elaphecarinata*	EN	39.9414	115.7889
Reptilia	Squamata	Colubridae	*Coluberspinalis*	LC	39.9382	115.8510
Reptilia	Squamata	Lacertidae	*Eremiasbrenchleyi*	LC	39.9414	115.7889

**Table 7.** Checklist of insects in Jingxi.

Species	Malaise trap	Pitfall traps	Convenience sampling by observation	Note
*Clinteroceramandarina* (Westwood, 1874)		y		
*Stictolepturasuccedanea* (Lewis, 1879)		y		
*Holotrichiatitanis* Reitter, 1902		y		
*Onthophagus* sp.		y		
*Maladeraorientalis* (Motschulsky, 1857)		y		
*Brahminafaldermanni* Kraatz, 1892		y		
*Pseudosymmachiaflavescens* (Brenske, 1892)		y		
*Maladera* sp.		y		
*Hemicrepidius* sp.		y		
*Selatosomus* sp.		y		
*Blaps* sp.		y		
*Oodescelispunctatissima* (Fairmaire, 1886)		y		
Carabus (Scambocarabus) sculptipennis Chaudoir, 1877	y	y		
*Carabusgranulatus* Linnaeus, 1758	y	y		
*Carabuscrassesculptus* Kraatz, 1881	y	y		
*Poecilusnitidicollis* Motschulsky, 1844	y	y		
*Agonumgracilipes* (Duftschmid, 1812)	y			
*Amaragigantea* (Motschulsky, 1844)	y			
*Harpaluscalceatus* (Duftschmid, 1812)	y			
*Carabusmanifestus* Kraatz, 1881	y	y		
*Opiloluteonotatus* Pic, 1926	y			
*Borboresthessubapicalis* Pic 1934	y			
*Bruchidiuscomptus* (Sharp, 1886)	y			
*Ocypusweisei* Harold, 1877	y			
*Agrilusviridis* (Linnaeus, 1758)	y			
*Liliocerisruficollis* (Baly, 1865)	y			
*Mordellistenatrifasciata* (Say, 1826)	y			Originated from North America, exotic / possibly invasive species
*Hemipyxisplagioderoides* (Motschulsky, 1861)	y			
*Pseudocneorhinushlavaci* Ren, Borovec & Zhang, 2019	y			
*Asiophridaxanthospilota* (Baly, 1881)	y			
*Serica* sp.	y			
*Borboresthesacicularis* Marseul, 1876	y			
*Smaragdina* sp.	y			
*Claddiscusobeliscus* Lewis, 1895	y			A new provincial record genus of Beijing
*Trachysaurifluus* Solsky, 1875	y			
*Cybocephalusnipponicus* Endrody-Younga, 1971	y			
*Caenocara* sp.	y			
*Micrambesinensis* Grouvelle, 1910	y			
*Ernobiusmollis* (Linnaeus, 1758)	y			
*Harmoniaaxyridis* (Pallas, 1773)	y			
*Falsomordellistena* sp.	y			
*Chlorophorussimillimus* (Kraatz, 1879)	y			
*Clerusdealbatus* (Kraatz, 1879)	y			
*Stigmatiumnakanei* Iga, 1949	y			
*Oenopiascalaris* (Timberlake, 1943)	y			
*Anapsis* sp.	y			
*Ectasiocnemisanchoralis* Nomura, 1961	y			
*Longitarsusdorsopictus* Chen, 1939	y			
*Byctiscusbetulae* (Linnaeus, 1758)	y			
*Magdalisfrontalis* (Gyllenhal, 1827)	y			
*Eumyllocerussectator* (Reitter, 1915)	y			
*Araecerus* sp.	y			
*Trachys* sp.	y			
*Camponotusjaponicus* Mayr, 1866	y			
*Chrysis* sp.	y			
*Sympiesis* sp.	y			
*Vulgichneumonleucaniae* (Uchida, 1924)	y			
*Aphidiusgifuensis* (Ashmead, 1906)	y			
*Aphidiusavenae* Haliday, 1834	y			
*Vespabicolor* Fabricius, 1787	y			
*Vespulaflaviceps* (Smith, 1870)	y			
*Megarhyssapraecellens* (Tosquinet, 1889)	y			
*Gasteruption* sp.	y			
*Heteribalia* sp.	y			
*Ammophila* sp.	y			
*Enicospilus* sp.	y			
*Nephrotomascalarisparvinotata* (Brunetti, 1918)	y			
*Contarinia* sp.	y			
*Aphidoletesaphidimyza* (Rondani, 1847)	y			
*Hemipenthesvelutina* (Meigen, 1820)	y			
*Muscadomestica* Linnaeus, 1758	y			
*Voriaruralis* (Fallen, 1810)	y			
*Episyrphusbalteata* (De Geer, 1776)	y			
*Ptecticusaustralis* Schiner, 1868	y			
*Cophinopodachinensis* (Fabricius, 1794)	y			
*Macrocera* sp.	y			
*Matronabasilaris* Selys, 1853			y	
*Mnais* sp.			y	
*Sympetrumeroticum* (Selys, 1883)			y	
*Aeshnamixta* Latreille, 1805			y	
*Papilioxuthus* Linnaeus, 1767			y	
*Pierisrapae* (Linnaeus, 1758)			y	
*Pontiadaplidice* (Linnaeus, 1758)			y	
*Coliaspoliographus* Motschulsky, 1860			y	
*Neptissappho* (Pallas, 1771)			y	
*Neptisrivularis* (Scopoli, 1763)			y	
*Childrenazenobia* (Leech, 1890)			y	
*Argyronomelaodice* (Pallas, 1771)			y	
*Polygoniac-aureum* (Linnaeus, 1758)			y	
*Polygoniac-album* (Linnaeus, 1758)			y	
*Loxerebiasaxicola* (Oberthür, 1876)			y	
*Minoisdryas* (Scopoli, 1763)			y	
*Everesargiades* (Pallas, 1771)			y	
*Celastrinaargiola* (Linnaeus, 1758)			y	
*Lycaeidesargyrognomon* (Bergsträsser, [1779])			y	
*Ochlodessubhyalina* (Bremer & Grey, 1853)			y	
*Vanessaindica* (Herbst, 1794)			y	
*Hestinaassimilis* (Linnaeus, 1758)			y	
*Libythealepita* Moore, [1858]			y	
*Notocryptacurvifascia* (C. & R. Felder, 1862)			y	
*Sericinusmontelus* Gray, 1852			y	
